# Extraction and Characterization of Gelatin from Skin By-Products of Seabream, Seabass and Rainbow Trout Reared in Aquaculture

**DOI:** 10.3390/ijms222212104

**Published:** 2021-11-09

**Authors:** Jesus Valcarcel, Carolina Hermida-Merino, Manuel M. Piñeiro, Daniel Hermida-Merino, José Antonio Vázquez

**Affiliations:** 1 Group of Recycling and Valorization of Waste Materials, Marine Research Institute (IIM-CSIC), Eduardo Cabello 6, 36208 Vigo, Spain; jvazquez@iim.csic.es; 2 Centro de Investigaciones Biomédicas (CINBIO), Departamento de Física Aplicada, Facultad de Ciencias, Universidade de Vigo, 36310 Vigo, Spain; manumar@uvigo.es; 3 Netherlands Organization for Scientific Research (NWO), DUBBLE@ESRF, BP220, F38043 Grenoble, France; daniel.hermida_merino@esrf.fr

**Keywords:** rainbow trout, seabass, seabream, gelatin, valorization

## Abstract

The expansion of fish filleting, driven by the increasing demand for convenience food, concomitantly generates a rising amount of skinning by-products. Current trends point to a growing share of aquaculture in fish production, so we have chosen three established aquaculture species to study the properties of gelatin extracted from their skin: rainbow trout, commonly filleted; and seabass and seabream, marketed whole until very recently. In the first case, trout skin yields only 1.6% gelatin accompanied by the lowest gel strength (96 g bloom), while yield for the other two species exceeds 6%, and gel strength reaches 181 and 229 g bloom for seabass and seabream, respectively. These results are in line with the proportion of total imino acids analyzed in the gelatin samples. Molecular weight profiling shows similarities among gelatins, but seabass and seabream gelatins appear more structured, with higher proportion of β-chains and high molecular weight aggregates, which may influence the rheological properties observed. These results present skin by-products of seabream, and to a minor extent seabass, as suitable raw materials to produce gelatin through valorization processes.

## 1. Introduction

Fish consumption has risen steadily in the last decades, accompanied by an increasing share of aquaculture production at the expense of wild capture [1]. In line with this trend, preferences of an ever wealthier and urbanized population are drifting towards convenience foods, pushing the fish industry from marketing whole fish to filleted products [2]. As a result, the generation of by-products from filleting aquaculture species can be expected to grow in the future, spurring the need for adequate management.

Industrial fish filleting represents a considerably wasteful practice, yielding only between 30 to 50% of muscle [3]. Such considerable amount of by-products requires realistic uses to increase resource efficiency and sustainability in fish production. Viable alternatives include recovery of edible parts from heads, frames, belly flaps and certain viscera [4]; oils rich in polyunsaturated fatty acids from heads, gills, and guts [5]; protein hydrolysates as food and feed ingredients from heads, trimmings and frames [6,7], and gelatin and collagen from both skin and bones [8,9,10]. Among these alternatives, gelatin and collagen extraction are particularly attractive because of their higher value compared with the other options [11,12].

Gelatin results from the partial denaturation of collagen, the main structural protein in connective tissue, rendering a more soluble material with wider applicability [13]. Gelatin properties depend on a number of factors such as animal species, age, source tissue, and extraction conditions [14]. In fish, the skin provides higher yield and quality gelatin than bone [15]. Therefore, skin by-products from filleting new species may represent a good source of gelatin not previously described because of lack of availability of the raw material.

With this view, we have selected two species marketed whole until very recently, seabass (*Dicentrarchus labrax*) and seabream (*Sparus aurata*); and the long-established rainbow trout (*Oncorhynchus mykiss*). In the case of seabass, a number of reports exist on gelatin and collagen from Asian seabass (*Lates calcarifer*) [16,17,18], but not from European seabass (*Dicentrarchus labrax*). In seabream, only one previous work deals with the extraction of gelatin from scales [19], but none characterizes gelatin from seabream skin.

In the present work, we use an established method to extract gelatin from the skin of seabass, seabream, and trout, all reared in aquaculture, followed by chemical and rheological characterization. Estimations of absolute molecular weight by gel permeation chromatography with light scattering detection, performed for the first time in these materials, allow to relate the physical properties of the gels with gelatin structure. This represents fundamental information for the design of valorization processes to recover gelatin from skin waste of the studied species.

## 2. Results and Discussion

### 2.1. Production of Gelatin from Seabream, Seabass and Trout Skin

To the best of our knowledge, no studies have explored the recovery of gelatin from the skin waste of seabream and European seabass, and research on rainbow trout is also quite limited. Thus, in the present work we study the process of gelatin extraction and determine the physicochemical properties of the gelatins obtained from these species.

Seabream produced the gelatin with the highest gel strength (230 g), closely followed by seabass (181 g), with differences statistically significant (*p* < 0.05). Yield for both species was similar (*p* > 0.05) with values around 7% *w*/*w* (Table 1). On the other hand, trout skin yielded a lower amount of gelatin (1.6% *w*/*w*) endowed with poorer jellification properties at 95 g (*p* < 0.05 in both cases).

Comparison with other research is far from straightforward, because both yield and gel strength depends on a number of variables such as chemical treatment, extraction temperature, or fish size and age [17,20,21]. Bearing this in mind, previous works have reported yields and gel strengths in rainbow trout much higher than in the present study, at 9.4% and 459 g respectively, possibly influenced by processing with higher concentrations of alkali and acid [20,21]. 

For seabream and seabass, direct comparison is not possible, as no previous studies could be retrieved on the extraction of gelatin from these species. However, a previous study extracted gelatin from the scales only, reaching higher yields and gel strength (12.9% and 312–317 g respectively) [19] than those reported here. This may be an interesting alternative when descaling is part of the process, but this is not generally the case in fish filleting.

In Asian seabass, previous studies report gelatin yield ranging from 43.5–66.4% (equivalent to 8.7 to 12.3% assuming 80% water content in the skin) and gel strengths from 233–369 g [17,22]. Interestingly, the gel strength of European seabass and seabream gelatin is closer to that of Asian seabass, an inhabitant of the warm waters in the Pacific-Indic oceans, than to cold water fish such as cod (90 g), pollock (98 g) or halibut (14 g) [13,23,24].

### 2.2. Characterization of Gelatin

#### 2.2.1. Amino acid Profiling

Table 2 displays the results of complete amino acid analysis of the three gelatin samples extracted. The percentage of the most important amino acids in a collagen derivative (OHPro is only present in the collagen protein) such as gelatin were 21.4–24.2%, 9.9–11.6% and 8.3–9.3% for glycine, proline and hydroxyproline, respectively. The sum of imino acids (Pro + OHPro) was in all cases higher than 18%, which is the minimum value for gelatin to have good texturometric properties [10]. The maximum content of those imino acids was significantly superior (*p* < 0.05) in seabream (20.4%), followed by seabass (19.9%) and trout (18.2%). In nutritional terms, the abundance of human essential amino acids in gelatins was always similar (around 26%), and regarding proximal composition, total protein and fat content were the highest (92.4%) and the lowest (3.4%) in seabream, respectively, and the lowest (84.6%) and the highest (9.4%) in rainbow trout.

Amino acid composition appears related to the gel strength of gelatins, with imino acids particularly relevant, since proline and hydroxyproline have been shown to stabilize gelatin gels [23]. Although differences in proline and hydroxiproline content among samples only range from 1 to 2% (9.90–11.11% Pro; 8.28–9.29 OHPro; Table 2), these small statistically significant differences may contribute to the relationship observed between gel strength and the sum of proline and hydroxyproline content. The imino acid content reported here for trout skin gelatin is higher than in a previous report (6.75% OHPro; 9.82% Pro) [24]. Unfortunately, gel strength was not determined in the latter, and no data exist for seabass and seabream. Some authors have suggested that other amino acids beyond the imino group may also contribute to the formation of triple helix structures from random coil, such as the hydroxylated amino acids threonine and serine [25], or alanine, predominant in non-polar regions [26]. While we found no relationship for the former in the present samples, alanine proportion is lower in trout than in the other two species (Table 2).

#### 2.2.2. Molecular Weight Distributions

**Figure 1:**
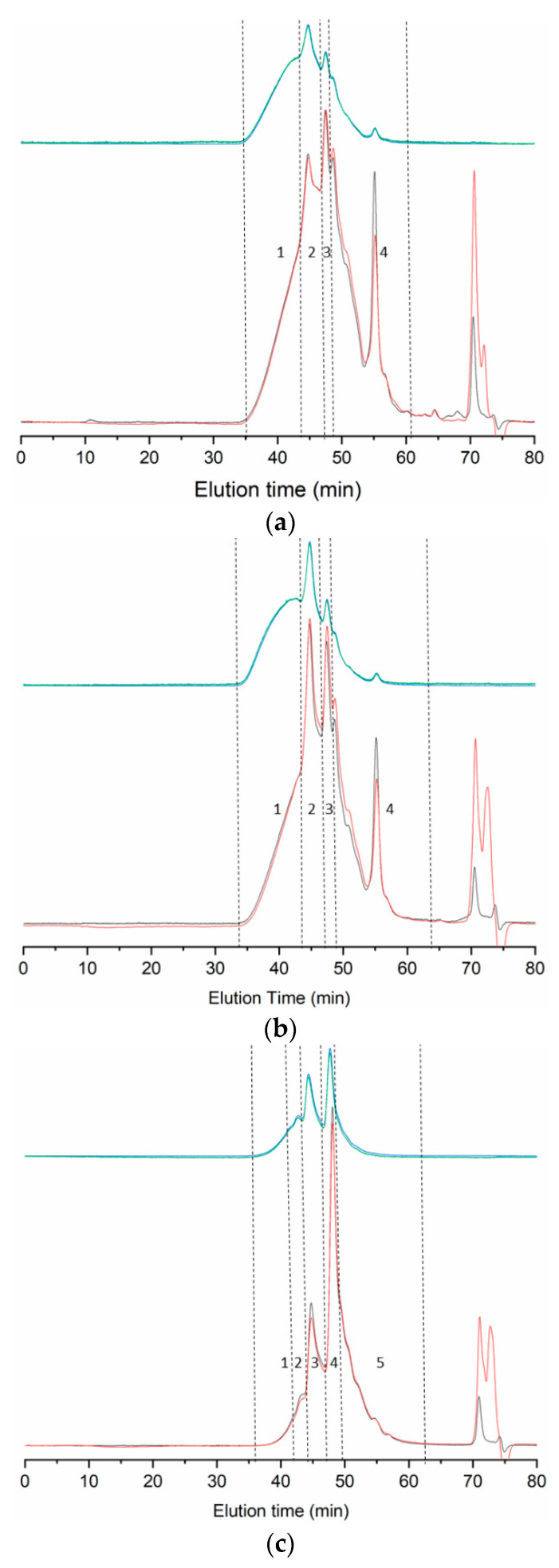
GPC eluograms of gelatin extracted from the skin of seabream (**a**), seabass (**b**), and trout (**c**). Blue line: right angle light scattering; green line: low angle light scattering; red line: refractive index; black line: ultraviolet (232 nm). Details about the numbered regions can be found in Table 2.

All gelatin samples present similar elution profiles (Figure 1), characterized by an initial high molecular weight fraction, followed by distinct peaks at around 45 and 48 min, and ending in a last fraction from 49 min onwards. An additional sharp peak appears in this last region at 55.6 min in seabream and seabass gelatin, but is virtually absent in trout.

Table 3 displays the absolute molecular weight of these regions estimated by two-angle light scattering. In high and low Mw species, estimates are not accurate because of the absence of distinct peaks, with the exceptions of the peaks at 43.8 min in trout (352 kDa) and at 55.6–55.7 min in seabream and seabass (43 and 32 kDa respectively). Molecular weight slightly surpasses 100 kDa for species eluting around 48 min in all samples, but differences exist for the earlier eluting peak: in trout, elution takes place at 44.8 min with calculated Mw of 207 kDa, whereas in seabass and seabream Mw reaches only 164–176 kDa, in line with longer elution times at 45.2 min. Remarkably, the polydispersity index (PDI) remains only slightly above 1 in all cases, indicating protein chains of similar length. 

The proportion of fractions 1–4, expressed as percentage area of the refractive index detector signal (Table 3), is very similar for seabream and seabass. The only difference lies in a slightly higher proportion of low Mw species in seabream (fraction 4). Interestingly, this low Mw fraction shows in trout (fraction 5) a comparable amount to seabass and seabream. Despite this similarity, the distribution of higher molecular weight fractions in trout gelatin is very different than in the other two fish. While seabass and seabream chains of 100 kDa comprise only 16.1–17.1% of the total area, this figures almost doubles in trout, rising to 30.2%. As expected, this goes in hand with a reduction of high Mw species from 17.5–20.5% in seabream and seabass to only 8.1% in trout.

The fraction slightly above 100 kDa found in all samples most likely corresponds to collagen α-chains, and the peaks of 207 kDa and 342 kDa seen in trout, to β-dimers and γ-chains (trimers), respectively. Species of intermediate Mw (164 kDa in seabream and 176 kDa in seabass) may consist of β-chains or partially degraded β-chains. The subtle differences in Mw between seabass and seabream do not allow to explain the differences seen in mechanical performance or amino acid profiles, maybe because these were also small. In the case of trout, gelatin shows less structuring than the other samples, mainly evident in the higher proportion of α-chains and lower amount of high Mw species. This is in spite of the presence of γ-chains in trout and the possible degradation of β-chains in seabass and seabream. 

Previous reports on the molecular weight of the species studied here are restricted to trout, with average Mw of around 300 kDa in gelatin extracted from the skin [20], and a maximum of 35% of β-chains [27]. Comparison with the first report is difficult, as the authors do not provide sufficient details about how the average Mw was calculated. In the second case, the amount of β-chains is 15% higher than the 20.3% reported here (Table 3), and was accompanied by considerably higher gel strength (459 vs. 95 g). 

#### 2.2.3. Thermal Stability

**Figure 2:**
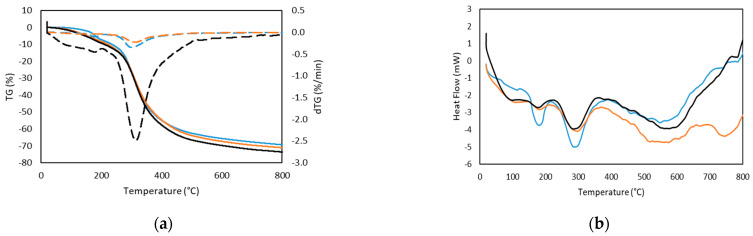
TGA thermogram (solid line, weight loss), and dTGA thermogram (dashed line, differential weight loss) obtained from TG curves (**a**); and DSC thermogram (**b**) of (blue line) seabass, (ochre line) trout and (black line) seabream.

The thermal stability of seabass, seabream and trout gelatins has been evaluated using the TGA thermogram and its derivative (Figure 2a). The curves show two zones of weight loss. First, a slight degradation step starting around 160 °C (Table 4), except for seabream gelatin which already shows a mass loss at 80 °C. However, this first stage leads to a weight variation of around −8% for seabass, seabream and trout gelatin. These mass changes up to 200 °C are related to the different forms of association of water with the structure of gelatin. Through hydrogen interactions, water can associate within and on the surface of the triple helix and associate between protein chains [28]. The next event occurred around 300 °C (Table 4),with a much more pronounced decrease in mass, which leads to a weight reduction of 60%; this loss of mass was related to the degradation of gelatin with the consequent loss of amino acids, as well as structurally bound water [29].

The thermogram (Figure 2b) shows several endothermic peaks at approximately 200 and 310 °C; a glass transition temperature (Tg) over 80 °C also appears, being more marked in the case of trout. The first peak may be related to the content of bound water that remains in the drying process and the adsorption of water during storage or with conformational changes in the structure of the protein [28]. The thermal events at about 300 °C may be associated with the gelatin degradation process, with loss of amino acids [29]. This temperature is in accordance with the thermogravimetric behavior (Figure 2a).

#### 2.2.4. Fourier Transform Infrared (FTIR) Spectra

**Figure 3:**
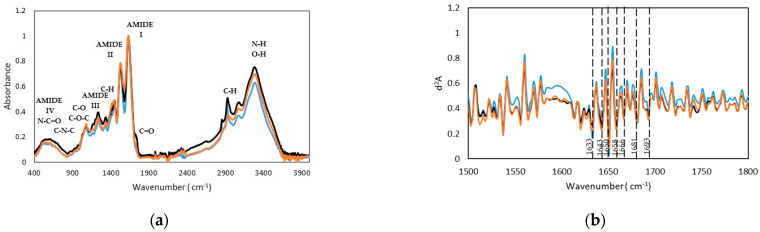
FTIR spectra (**a**) and spectra of the second derivative (differential FTIR spectra, with FDD method) in the absorption region of amide I (**b**), of (**blue line**) seabass, (**ochre line**) trout and (**black line**) seabream gelatins.

The FTIR spectra presented in Figure 3a display the main bands typical of gelatin, with specific wavenumbers and assignments compiled in Table 5. First, an intense and broad band appears around 3280 cm^−1^, due to the tension modes of the N-H bonds of the protein and O-H groups of carbohydrates and water. The signals observed between 2850 and 3000 cm^−1^ correspond to the tension modes of the C-H bonds of aliphatic chains, and the flexion modes are between 1333 and 1454 cm^−1^. Amide bands I, II and III appear at 1630, 1525 and 1235 cm^−1^, and the other less intense bands that emerge around 520, 600, 700 cm^−1^ are due to amide bands IV, V and VI respectively. Above 975 cm^−1^, a weak band appears corresponding to the symmetric tension mode of the CNC bond. Bands at 1160 and 1080 and 1030 cm^−1^ correspond to different tension modes of C-O and C-O-C bonds that could be due to carbohydrates. The rounded and broad shape of the band around 600 cm^−1^ is due to the presence of water in the sample. In trout gelatin, a band appears at 1720 and 1737 cm^−1^ corresponding to the C=O of lipid residues, that is non-existent for seabass and seabream gelatins.

The absorption in the amide I region (1620–1700 cm^−l^) is the most useful for infrared spectroscopic analysis of the secondary structure of proteins. To obtain information about the secondary structure of protein, the FTIR spectra of gelatin were analysed using the second derivative method in the absorption region of the Amide I (see Figure 2b).

By comparison with the literature [30,31], the 1633 cm^−l^ peak can be associated with the β-segments. The weak peaks about 1700 cm^−1^ in trout gelatin correspond to the second β-structure band and to turns [30,32]. The 1680 cm^−1^ band can be associated with the β-strands, while the 1666 and 1690 cm^−1^ bands are due to turns [30]. The 1650 cm^−l^ (weak) and 1633 cm^−1^ (strong) peaks are evidently associated with these substructure classes, the 1668 to 1693 cm^−1^ bands, with the second β-structure band and with turns [30]. Furthermore, amide III bands from seabass, seabream and trout gelatin hydrogels were observed at the wavenumber of 1235–1236 cm^−1^, which indicates disorder in the gelatin molecule and is more likely related to loss of the triple helix state [22].

Quantitative analysis of the amide I band contour was done using the deconvolution method. The amide I band, between 1600 and 1700 cm−1, is the most useful for infrared spectroscopic analysis of the secondary structure of proteins. The spectral range decomposes into five bands. The fractions of each component in the resolution-enhanced amide I band were estimated quantitatively by a nonlinear least-squares fitting program Fityk [33]. The deconvoluted spectrum was fitted with Gaussian band shapes by an iterative curve fitting procedure, that is, the proportion of each component in the amide I band is calculated as the area fraction of the corresponding peak divided by the sum of the areas of the peaks that belong to the amide I band [34].

**Figure 4:**
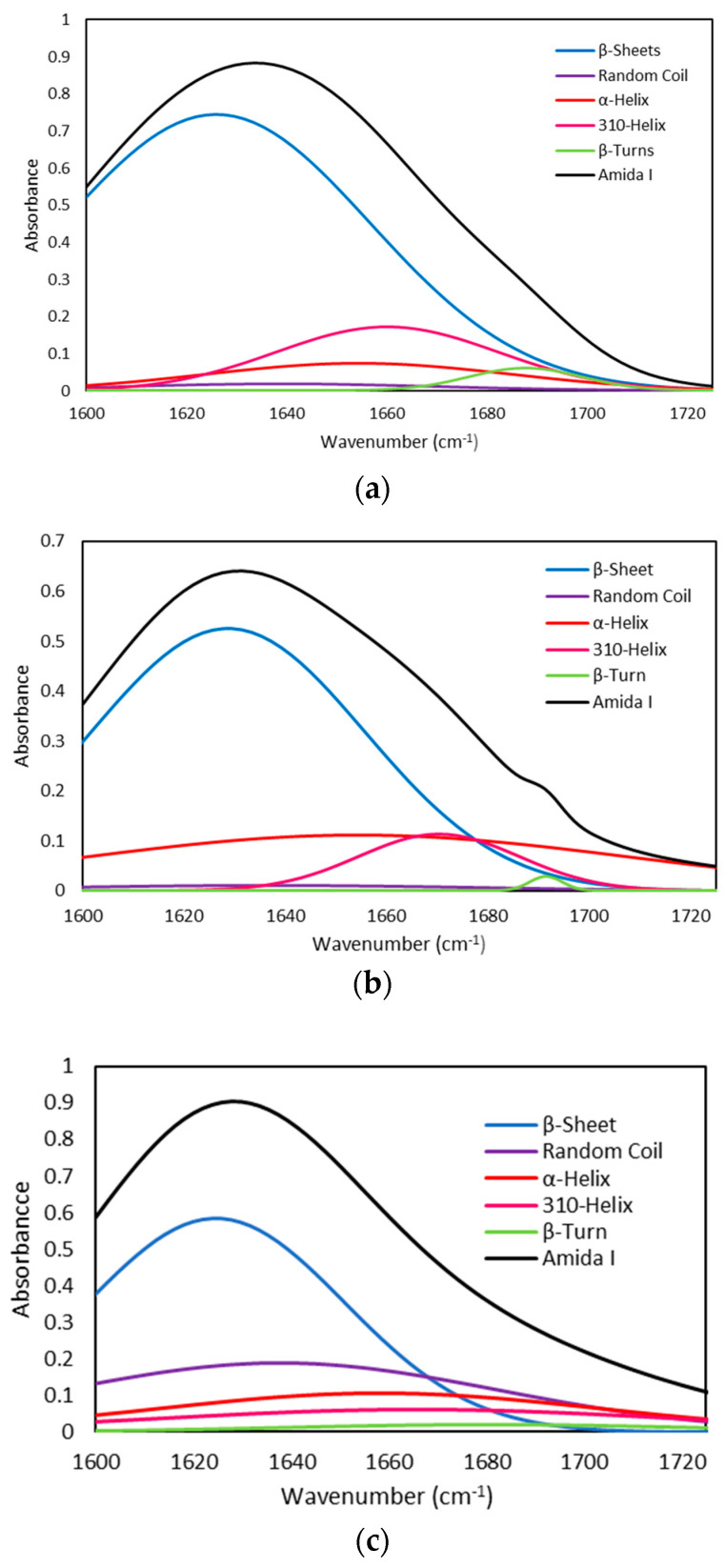
Amide I band decomposition by using Gaussian distribution. (**a**) Seabass, (**b**) Seabream, and (**c**) Trout gelatin.

Figure 4 shows the deconvoluted spectra for the amide I bands of seabass, seabream and trout gelatin, observing that they are rich in beta-structure. Table 6 presents the observed frequencies, vibrational assignments, and % areas for each amide I band component of seabass, seabream and trout gelatin.

#### 2.2.5. Rheology

**Figure 5:**
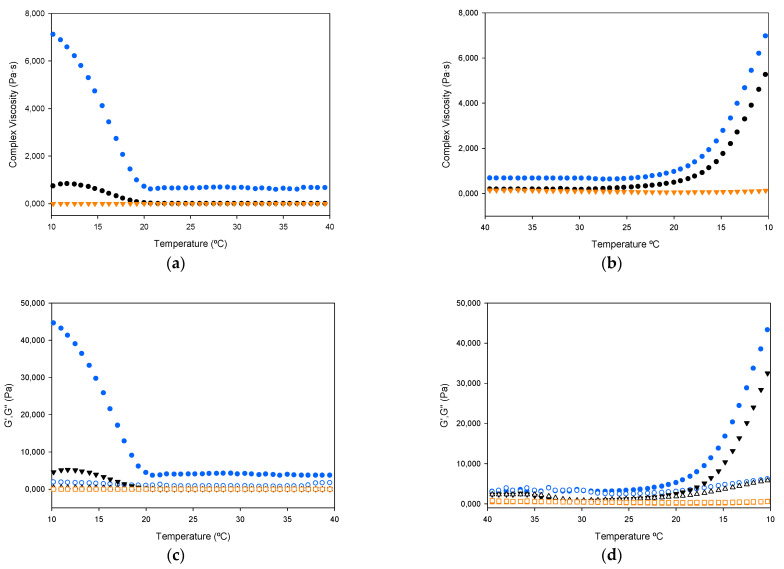
Variation of complex viscosity (**a**,**b**); and storage modulus (G′, filled symbols) and loss modulus (G″, empty symbols) (**c**,**d**); gelatin hydrogels (30% wt in water) with temperature. Heating ramp (left column) from 10 to 40 °C, cooling ramp (right column) from 40 to 10 °C. (blue symbol) seabass, (ochre symbol) trout and (black symbol) seabream gelatins.

The study of the viscoelastic properties of gelatin started by analyzing the thermal transitions of gelatin hydrogels (30% wt gelatin in water) during heating (10–40 °C) and cooling (40–10 °C), which provides information about gelling kinetics, namely complex viscosity, storage, and loss moduli. As seen in Figure 5, no marked differences in gelation temperature exist between the different gelatin hydrogels, with similar values to others found in the literature [22]. In all cases a rapid transition occurs, developing a strong gel matrix. 

For the three gelatin hydrogels, the complex viscosity increases with decreasing temperature (cooling ramp) and decreases again with increasing temperature (heating ramp). Generally speaking, a higher viscosity helps to maintain the integrity of the gel formulation, and the SOL-GEL transition below the critical gel temperature makes it more suitable for preparing more complex structures with good shape printability [35].

As the temperature decreased, the G′ and G″ of the seabass and seabream gelatin solution increase sharply, more pronounced in the case of seabass, indicating the formation of a gel. The gelling process can be attributed to the physical crosslinking of single chains (connecting regions or interactions, including hydrogen bonds, ionic interactions, van der Waals forces, self-assembly, and hydrophobic associations). The gelation temperatures of all gelatin samples were in the range of 19.5 to 20 °C, which is similar to the typical gelling point range of fish gelatin [35].

Furthermore, upon cooling, a crossover occurred for G′ and G″,assigned as the “GEL point” or “SOL-GEL” transition temperature, indicating the transition from a solution state to a gel stateas the temperature decreased. The temperature at which the curves of G′ and G″ crossover of, also known as sol-gel transition temperature, is defined as a gelling point (phase transition temperatures) (gelation points, G′ = G″) [36].

Seabass gelatin hydrogel had the highest gelling ability, showing a significantly higher increase in G′ and G″ upon cooling than seabream and trout gelatin hydrogels, which had quite similar behaviour (Figure 5a,b). These differences also appear in the viscoelastic properties upon heating from 10 to 40 °C. Whereas both seabream and trout gelatin form hydrogels in a similar way, seabass gelatin showed noticeably higher G′ and G″ values (Figure 5c,d) [37]. The literature reports similar values for the gelling and melting temperatures [38].

**Figure 6:**
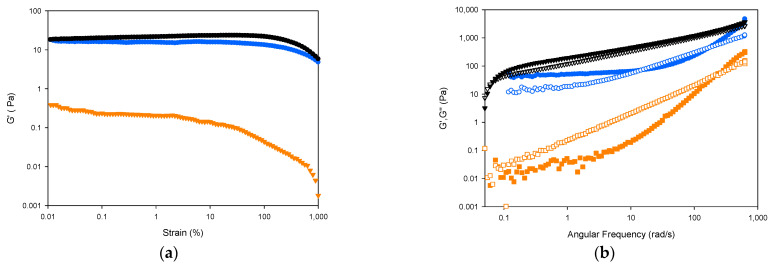
Strain and frequency sweeps for solutions of seabass, trout and seabream gelatins (30% wt in water). Store (G′); (filled symbols) and loss moduli (G″); (hollow symbols) depicted versus strain (**a**) and angular frequency (**b**). (blue symbol) seabass, (ochre symbol) trout and (black symbol) seabream gelatin hydrogels at 20 °C.

Strain sweeps allow for the evaluation of the viscoelastic behavior of gelatins by determining the range where their rheological properties are independent of the applied deformation. Strain sweeps of 30% solutions of seabass, trout and seabream gelatin hydrogels (Figure 6a) show broadly typical gel behaviour, with a plateau region in the module value with low strain value and, finally, significant drops in the module values. 

The strength of the gel network can be evaluated by studying the frequency dependence of the modulus. Figure 6b shows the mechanical spectra of gelatin solutions at 20 °C in terms of G′ and G″ as a function of frequency. Seabass and seabream gelatin hydrogels show a gel-like behaviour, as denoted by G′ > G″ values. The elastic modulus G′ was clearly lower in trout gelatin hydrogel as compared to gelatin from seabass and seabream [37]. The seabream gelatin hydrogel shows the highest value for the storage and loss modulus, as a result of higher concentrations of helical structures, followed by slightly lower but similar values in seabass gelatin hydrogel. For seabream and trout gelatin hydrogels, in the higher frequency range analyzed, G″ crosses G′, which means that a relaxation zone begins near the transition of the colloidal gel, increasing G″ significantly, which indicates higher gel strength [39].

## 3. Conclusions

The extraction of gelatin from seabass and seabream appears as a suitable strategy for the valorization of filleting skin by-products. The physicochemical properties of the gelatin produced from both species are quite similar, with gel strength lying halfway between cold and warm water fish species. On the other end, trout gelatin shows weaker rheological properties, which reflect a lower amount of imino acids and higher proportion of low molecular weight species. 

While seabass, seabream, and trout gelatin do not match the properties of traditional gelatin from terrestrial animals, these fish gelatins may find new applications, such as in the formulation of foods when fast dissolution in the mouth is required [40], as gel-sol transition takes place around 21 °C in all gelatins; or in the microencapsulation of liposoluble vitamins, performed with gelatins of up to 140 g [9], where trout gelatin (98 g) could be of use. Furthermore, incorporation of fish gelatin into composite materials and chemical modification of fish gelatin may tune its properties to match a range of other applications, such as tissue engineering, drug delivery, wound dressing, food packaging, and 3D printing [41,42].

## 4. Materials and Methods

### 4.1. Skin By-Products from Aquaculture Fish

The skins of aquaculture species seabream (*Sparus aurata*), seabass (*Dicentrarchus labrax*) and rainbow trout (*Oncorhynchus mykiss*) were produced after filleting fish obtained from a local market in Vigo (Galicia, Spain). The waste material was stored at −20 °C until further use. Each skin was cut in fragments less than 5 × 5 cm and 500 g of these fragments were processed per batch. In all cases, the first step was a washing water step for 30 min under orbital agitation (50 rpm) to eliminate the impurities present in the skin portions.

### 4.2. Extraction of Aquaculture Gelatin

For each species, the steps employed for the production of fish gelatins were based on the protocol defined by [43] with some modifications. The first stage was the alkaline treatment of clean skins with 0.05 M NaOH using a solid:liquid ratio of (1:4) for 30 min under 50 rpm of orbital agitation at 22 °C. This procedure was repeated to increase the extraction of fat present in the skins. Chemical effluents were filtered (1 mm) and the skins were washed in water for 30 min. Subsequently, the skins were treated with 0.02 M H_2_SO_4_ at 22 °C, with (1:4 ratio) for 30 min, under 50 rpm of agitation. After the corresponding water wash, 0.052 M citric acid (1:4 ratio, 30 min, 22 °C and 50 rpm) was applied for the final chemical processing of skins. Solutions of gelatin were obtained by thermal extraction at 45 °C on aqueous medium (1:2 ratio) for 16 h. For their purification, gelatin solutions were filtered through 500 µm, mixed with active charcoal (1.5% *w*/*v*) for 2 h and centrifuged (15,000× *g*/20 min) for the elimination of the charcoal and impurities. Finally, solid gelatins solutions were dried in an oven for 48–72 h. For each fish species, procedures for gelatin production were executed in duplicate. 

### 4.3. Yield and Amino acid Profile

Production yields were calculated as the dry weight (g) of gelatin extracted per 100 g of fresh skins before processing. Total protein and total lipids were analyzed in dry gelatins as total nitrogen × 6.11 [43,44] and by the gravimetric method after Soxhlet extraction using reflux and diethyl ether as solvent [45], respectively. Amino acid content was quantified by ninhydrin reaction [46] employing an amino acid analyser (Biochrom 30 series, Biochrom Ltd., Cambridge, UK) and norleucin as internal standard. 

### 4.4. Molecular Weight

The molecular weight profiles of aquaculture gelatins were analyzed by gel permeation chromatography with an Agilent 1260 LC system (Agilent, Santa Clara, CA, USA) consisting of quaternary pump (G1311B), injector (G1329B), column oven (G1316A), DAD (G1315C) refractive index (G1362A) and dual angle static light scattering (G7800A) detectors. Proteema precolumn (5 µm, 8 × 50 mm), Proteema 100 Å (5 µm, 8 × 300 mm), Proteema 300 Å (5 µm, 8 × 300 mm) and Proteema 1000 Å (5 µm, 8 × 300 mm) (PSS, Mainz, Germany) were used for polymer separation. The system was kept at 20 °C and 0.15 M sodium acetate: 0.2 M acetic acid, pH 4.5 was used as mobile phase, at a rate of 0.5 mL/min. Samples were dissolved at 1.8–2.2 g/L in the GPC mobile phase. To avoid errors due to incomplete dissolution of samples, a refractive index increment (dn/dc) of 0.190 [47] was used to estimate the molecular weight. 

### 4.5. Gel Strength

The strength of gelatin gels were measured by the method detailed in [48]. Briefly, solutions of gelatin were prepared at a concentration of 6.67% (*w*/*v*), completely dissolved at 45 °C and cooled at 4°C for 16–18 h [49]. Gel strength was measured using a Stevens-LFRA Texture Analyzer (Hucoa Erlöss S.A., Madrid, Spain) with a 1000 g load cell equipped with a 0.5 inch of diameter Teflon probe. A trigger force of 5 g and a penetration speed of 1 mm/s were used and gel strength was expressed as maximum force (in g), taken when the plunger had penetrated 3 mm into the gelatin gels, as average of three determinations. 

### 4.6. Fourier Transform Infrared (FTIR) Spectroscopy

Fourier transformed infrared spectroscopy by attenuated total reflectance (ATR-FTIR) was obtained using a spectrometer (Nicolet 6700, Thermo Fisher Scientific, Waltham, MA, USA), equipped with a source IR-Turbo fitted with a detector DTGS in a beamsplitter of KBr. The frequency range was from 4000 to 400 cm^−1^. Background scans were 34 with a spectral resolution of 4 cm^−1^ at ambient temperature, using an attenuated total reflectance (ATR) accessory. Seabass, seabream and trout gelatins were deposited in a gold support in a humid chamber to avoid evaporation during measurements. Analysis of the secondary structure of gelatin was done using the second derivative of the spectra of Amide I, using the first difference derivative (FDD method).

### 4.7. Thermal Stability

Thermogravimetric analysis (TGA) of seabass, seabream and trout gelatins were performed with a Setsys Evolution 1750 simultaneous thermogravimetric analysis (TGA)/differential scanning calorimetry (DSC) instrument (Setaram, Caluire-et-Cuire, France). Sample sizes ranging from 10 to 13 mg were introduced in a sealed capsule, undergoing temperature sweeps from room temperature to 600 °C at a heating rate of 5 °C/min^−1^, under an inert nitrogen atmosphere to avoid oxidation, and continuing at 900 °C under an air atmosphere to promote oxidation. The onset decomposition temperature and weight loss profiles were obtained.

### 4.8. Rheology

The rheological properties were measured using a Physica MCR 101 rheometer (Anton Paar, Graz, Austria) equipped with a conical plate geometry (CP50-1) with a constant space of 0.048 mm used to measure the strain and frequency sweep; the resistant plate (PP50/S) was 50 mm and the gap was 0,1 mm, which allowed for the control of the torque between 0.5 μN·m and 125 μN·m used to measure the temperature ramps [50]. The linear viscoelastic range was determined by performing a strain sweep from 0.1 to 1000% at a constant angular frequency of 10 rad/s at 20 °C, for the gelatins dissolved in distilled water (30%wt gelatin), forming a seabass, seabream and trout gelatin hydrogels. The solutions were preheated at 60 °C for 30 min for all rheological experiments. The storage modulus G′ and loss modulus G″ were determined in the range of linear deformation. Frequency sweep measurements were made from 0.05 to 600 rad/s, applying a constant 0.1% strain at 20 °C. For temperature ramps, the samples were heated from 10 to 40 °C and cooled from 40 to 10 °C to study gelatin gelation and subsequent melting.

## Figures and Tables

**Table 1:** Yield and gel strength of gelatin extracted from seabream, seabass and rainbow trout skin. Values expressed as average ± intervals of confidence for *n* = 2 (replicates of independent batches) and α = 0.05. Different superscript letters in each row mean statistically significant differences between fish species (*p* < 0.05).

Species	Yield (%, w of Gelatin/w of Skin)	Gel Strength (Bloom, g)
Seabream	6.60 ± 0.46 ^a^	229.5 ± 12.7 ^a^
Seabass	6.83 ± 0.39 ^a^	181.0 ± 7.8 ^b^
Trout	1.56 ± 0.24 ^b^	95.0 ± 2.0 ^c^

**Table 2:** Amino acid (AA) content of gelatins recovered from fresh aquaculture fish skins (% or g/100 g total amino acids) with each protocol of production. OHPro: hydroxyproline. Pr: % of protein present, as the sum of amino acids, in the extracted gelatin sample. TE/TA: ratio total essential amino acids for human/total amino acids. Errors are the confidence intervals for *n* = 2 (replicates of independent batches) and α = 0.05. Different superscript letters in each row mean statistically significant differences between fish species (*p* < 0.05).

AA	Seabream	Seabass	Trout
Asp	5.51 ± 0.15	5.98 ± 0.05	6.31 ± 0.05
Thr	2.94 ± 0.18	3.02 ± 0.02	2.75 ± 0.02
Ser	4.32 ± 0.08	4.58 ± 0.20	4.94 ± 0.09
Glu	9.46 ± 0.03	10.15 ± 0.12	10.03 ± 0.10
Gly	23.02 ± 0.38 ^a^	21.42 ± 0.29 ^b^	24.16 ± 0.26 ^c^
Ala	10.18 ± 0.02	10.59 ± 0.12	9.14 ± 0.07
Cys	0.37 ± 0.02	0.27 ± 0.02	0.41 ± 0.08
Val	2.08 ± 0.15	1.97 ± 0.06	1.81 ± 0.05
Met	2.41 ± 0.27	2.05 ± 0.01	3.09 ± 0.09
Ile	1.01 ± 0.04	0.99 ± 0.03	1.41 ± 0.02
Leu	2.52 ± 0.05	2.49 ± 0.07	2.59 ± 0.08
Tyr	0.73 ± 0.09	0.70 ± 0.09	0.70 ± 0.05
Phe	2.24 ± 0.20	2.41 ± 0.05	2.17 ± 0.04
His	0.78 ± 0.14	0.80 ± 0.05	1.04 ± 0.27
Lys	3.70 ± 0.01	3.93 ± 0.21	3.52 ± 0.13
Arg	8.33 ± 0.00	8.75 ± 0.18	7.76 ± 0.07
OHPro	9.29 ± 0.37 ^a^	8.34 ± 0.37 ^b^	8.28 ± 0.54 ^c^
Pro	11.11 ± 0.19 ^a^	11.56 ± 0.01 ^b^	9.90 ± 0.60 ^c^
Pr (%)	92.4 ± 4.4 ^a^	89.9 ± 4.4 ^ab^	84.6 ± 3.1 ^b^
TE/TA (%)	26.1 ± 0.1 ^a^	26.4 ± 0.6 ^a^	26.1 ± 0.1 ^a^

**Table 3:** Molecular weight (kDa) of gelatin distributions from trout, seabass, and seabream shown in Figure 1. Rt: retention time; Mw: weight average molecular weight; PDI: polydispersity index. Peak area (%) corresponds to refractive index detector. Values are represented as mean ± standard deviation (*n* = 2).

	Peak Number	Rt (min)	Mw (kDa)	PDI	Peak Area (%)
Seabream	1-high Mw	35.2–44.02	-	-	17.5 ± 6.0
	2	45.2 ± 0.1	164 ± 7	1.016	26.1 ± 1.8
	3	48.0 ± 0.1	112 ± 10	1.003	16.1 ± 2.4
	4-low Mw	49.1–60.2	<100	-	32.8 ± 2.0
		55.6 ± 0.1	43 ± 7	1.002	7.5 ± 1.4
Seabass	1-high Mw	34.2–44.1	-	-	20.5 ± 4.2
	2	45.2 ± 0.0	176 ± 2	1.016	27.0 ± 0.2
	3	47.9 ± 0.0	112 ± 2	1.004	17.1 ± 1.4
	4-low Mw	49.1–64.22	<100	-	27.3 ± 3.0
		55.7.0 ± 0.0	32 ± 4	1.001	8.1 ± 1.2
Trout	1-high Mw	35.8–42.3	-	-	3.2 ± 0.8
	2	43.8 ± 0.1	342 ± 49	1.012	4.9 ± 0.3
	3	44.8 ± 0.1	207 ± 12	1.023	20.3 ± 3.7
	4	48.1 ± 0.0	117 ± 5	1.006	30.2 ± 3.8
	5-low Mw	49.1–62.5	<100	-	41.4 ± 8.6

**Table 4:** Values obtained from TGA and DTGA analysis of seabass, seabream and trout. Onset temperatures, T_onset_; maximum temperatures, T_max_; and Weight loss (%) at 5 °C/min.

	Steps	T _Onset_ (°C)	T_max_ (°C)	Weight loss (%)
Seabass	1	158.86	257.48	−8.07
	2	180.03	301.34	−61.46
Seabream	1	36.44	88.20	−9.46
	2	255.67	315.22	−64.62
Trout	1	44.53	254.58	−8.53
	2	112.90	312.46	−62.70

**Table 5:** Seabass, seabream and trout gelatins feature the following bands.

Wavenumber (cm^−1^)			Assignment
Seabass	Seabream	Trout	
3284	3277	3272	N-H/O-H
2850–3000	2850–3000	2850–3000	Aliphatic Tension CH
1333, 1444	1334, 1445	1338, 1454	Aliphatic Flexion CH
1632, 1525, 1236	1631, 1530, 1235	1628, 1529, 1236	Amide I,II,III
973	974	-	CNC
521, 601, 706	560, 603, 702	532, 607, 704	Amide IV,V,VI
1162–1077	1172, 1079, 1034	1161, 1078, 1032	Bond Tension C-O, C-O-C
600	600	600	Water
-	-	1720, 1737	Lipid C=O

**Table 6:** Location and percent area contribution of the main secondary structures in the amide I region of seabass, seabream and trout gelatin. FWHM: Full width at half its maximum value, corresponding to the widths of the central peaks of the autocorrelation traces.

Gelatin Type	Peak center (cm^−1^)	Area %	Height	FWHM	Assignment
Seabass	1629	57.35	0.74	72.42	β-Sheet
	1639	1.40	0.02	73.00	Random Coil
	1654	5.69	0.07	72.2	α-Helix
	1660	9.23	0.17	52.35	3_10_-Helix
	1687	1.77	0.06	26.62	β-turn
Seabream	1628	35.60	0.53	63.60	β-Sheet
	1636	1.13	0.01	105.80	Random Coil
	1654	14.99	0.11	127.28	α-Helix
	1669	4.52	0.11	37.19	3_10_-Helix
	1689	0.23	0.03	7.54	β-turn
Trout	1624	38.51	0.58	62.06	β-Sheet
	1637	20.63	0.18	105.49	Random Coil
	1658	10.67	0.09	106.6	α-Helix
	1666	11.69	0.09	124.8	3_10_-Helix
	1681	2.15	0.02	96.2	β-turn

## Data Availability

Not applicable.
